# Structural and Diffusion MRI Analyses With Histological Observations in Patients With Lissencephaly

**DOI:** 10.3389/fcell.2019.00124

**Published:** 2019-07-11

**Authors:** Lana Vasung, Arthur Rezayev, Hyuk Jin Yun, Jae W. Song, Andre van der Kouwe, Natalie Stewart, Arthi Palani, Tadashi Shiohama, Francois Chouinard-Decorte, Jacob Levman, Emi Takahashi

**Affiliations:** ^1^Division of Newborn Medicine, Department of Medicine, Boston Children’s Hospital, Harvard Medical School, Boston, MA, United States; ^2^Fetal Neonatal Neuroimaging and Developmental Science Center, Boston, MA, United States; ^3^Division of Neuroradiology, Department of Radiology, Massachusetts General Hospital, Harvard Medical School, Boston, MA, United States; ^4^Athinoula A. Martinos Center for Biomedical Imaging, Department of Radiology, Massachusetts General Hospital, Boston, MA, United States; ^5^Department of Pediatrics, Graduate School of Medicine, Chiba University, Chiba, Japan; ^6^Ludmer Centre for Neuroinformatics, McGill Centre for Integrative Neuroscience, Department of Biomedical Engineering, Montreal Neurological Institute and Hospital, McGill University, Montreal, QC, Canada; ^7^Department of Mathematics, Statistics, and Computer Science, St. Francis Xavier University, Antigonish, NS, Canada

**Keywords:** lissencephaly, gyrification, LIS1, DCX mutation, MRI, DTI

## Abstract

The development of cortical convolutions, gyri and sulci, is a complex process that takes place during prenatal development. Lissencephaly, a rare genetic condition characterized by the lack of cortical convolutions, offers a model to look into biological processes that lead to the development of convolutions. Retrospective, qualitative, and quantitative analyses of structural magnetic resonance imaging (MRI) and diffusion tensor imaging (DTI) were performed in patients with lissencephaly (*N* = 10) and age-/sex-matched controls (*N* = 10). In order to identify microstructural correlates of structural MRI and DTI findings, postmortem brains of patients with lissencephaly (*N* = 4) and age-matched controls (*N* = 4) were also examined with histology. Patients with lissencephaly had significantly smaller gyrification index and volumes of hemispheric white and gray matter, compared to the age-/sex-matched control group. However, there was no significant difference between groups in the subcortical gray matter volumes. Although the majority of patients with lissencephaly had a preserved normal-like appearance of major fissures and primary sulci, the spatial distribution of agyric cortical regions was different in patients with *lissencephaly-1* (*LIS1*) and *doublecortin* (*DCX*) mutations. Lastly, in patients with lissencephaly, the spatiotemporal distribution of projection pathways was preserved while short- to medium-range cortico-cortical pathways were absent or fewer in number. Our results indicate that in the patients with lissencephaly cortical system is affected more than the subcortical one.

## Introduction

Lissencephaly is a rare genetic condition characterized by a lack of cortical convolutions. It has been associated most frequently with the mutations of the *lissencephaly-1* (LIS1) (Reiner et al., 1993), *doublecortin* (DCX) (des Portes et al., 1998), and *tubulin alpha 1A* (TUBA1A) genes (Poirier et al., 2007). At the cellular level, lissencephaly results from insufficient neuronal migration. LIS1 lissencephaly is characterized by the “four-layered cortex” while in DCX lissencephaly the cerebral cortex displays six-layers (Viot et al., 2004). This is important because the appearance of cortical convolutions has been linked to the intensity of neurogenic processes such as the development of radial glial scaffolding (Fernández et al., 2016) used for radial migration of neurons (Rakic, 1972), differences in the rate of increasing thickness of supragranular (I–III) vs. infragranular layers (V–VI) (Armstrong et al., 1991), sequential cortical ingrowth of fibers (Kostovic and Rakic, 1990), increases in brain volume (Zilles et al., 2013), and accelerated growth of white compared to gray matter, reflecting the development of connectivity (Herculano-Houzel et al., 2010). Recently, Di Donato et al. (2018) reported differences in the phenotype between subjects that have mutations in single lissencephaly-associated gene. For example, mutations of LIS1 gene are associated with a higher occurrence of posterior pachygyria (sulci 1.5–3 cm apart with abnormally wide gyri) or posterior agyria–pachygyria. In contrast, mutations of the DCX gene are more frequently characterized by a diffuse agyria or predominantly frontal pachygyria. Therefore, identifying genotype–phenotype relations in lissencephaly can provide insights into the biological processes that drive the development of cortical convolutions (Dobyns, 1987).

The appearance of cortical convolutions is evident during the second half of pregnancy and it is accompanied by a 50-fold increase in the surface area of the cerebral cortex (Vasung et al., 2016). During this period, the ratio of the cortical volume (cortical plate and subplate) to the white matter (WM) volume decreases from approximately three to one, while the thickness of the cortical plate increases by less than a millimeter (Vasung et al., 2016; Andescavage et al., 2017). Since all of the neurogenic processes that occur during the second trimester can be disrupted in lissencephaly, our aim will be to quantitatively determine differences in gyrification and volumetric measurements between a heterogeneous group of patients and age-matched controls, as well as to qualitatively observe which measures may be associated with the absence of cortical convolutions.

## Materials and Methods

### Subjects

#### *In vivo* Subjects

Since this study was retrospective in nature and no identifying information would be requested by the researchers, it was determined that the informed consent would not be sought from the patients due to the low risk this study would present to them. Thus, a waiver of consent, in addition to all other procedures, was approved after due consideration by the Boston Children’s Hospital (BCH) Institutional Review Board. We retrospectively reviewed 14 MRI examinations from 10 patients with lissencephaly (0.68–21.67 years; Supplementary Tables S1–S3), and compared them with 10 age-matched neurologically healthy control subjects. All patients and control subjects were male.

#### *Ex vivo* Specimens

Coronal blocks of specimens with lissencephaly (*N* = 3) and the coronal blocks of age and sex-matched post-mortem control brains (*N* = 4) were obtained from the Brain and Tissue Bank for Developmental Disorders supported by the Eunice Kennedy Shriver National Institute of Child Health and Development^1^ (Supplementary Table S4). The coronal brain blocks were approximately 2 cm thick. Age, cause of death, brain malformation, and additional information can be found in Supplementary Table S2. After the successful MR scan, tissue blocks were cut and histological slices were stained with neurofilament staining that has previously been reported to be successful in the staining of axonal fibers of immature fetal brains (Widjaja et al., 2010).

### MRI Analysis

#### MRI Acquisition

##### In vivo MRI acquisition

Participants were imaged with a clinical 3T or 1.5T MRI scanner (Skyra, Siemens Medical Systems, Erlangen, Germany) at BCH, yielding T1 and T2 structural MRI and DTI. Due to the retrospective nature of the study, there was variability in the MRI parameters employed (Supplementary Table S1).

##### Ex vivo MRI acquisition

The blocks of postmortem brain were prepared for MRI scanning by soaking in Ziploc bags containing Fomblin oil (same used as, e.g., Takahashi et al., 2012). These bags were placed parallel to each other in a container, separated by plastic boards. The scans were performed at the Athinoula A. Martinos Center for Biomedical Imaging. Diffusion-weighted data were acquired using a 3 Tesla Siemens TIM Trio MRI machine with a 32 channel head coil, a 100 Hz/px bandwidth, and steady-state free-precession (SSFP)-based diffusion sequence. Each slab was 192 mm thick. Following parameters were used for SSFP: TR/TE = 24.82/18.76 ms, *a* = 60°, nex = 2, in-plane imaging matrix = 200 × 200, in-plane field of view = 160 × 160 mm^2^, in-plane imaging resolution 0.8 × 0.8 mm^2^, and slice thickness = 0.8 mm without a gap. Diffusion weighting was performed along 44 directions (*b* = 800 s/mm^2^) with 4*b* = 0 images. The directions used in the imaging were generated via electrostatic repulsion on the surface of a sphere to ensure equidistant spacing with some approximations. The total scan time was 17 h 35 min 24 s. When using SSFP, it is difficult to achieve a much higher *b*-value due to long scan time and constraints on gradient heating. The use of SSFP is relatively new but is effective when a high-resolution *ex vivo* diffusion is required.

##### Qualitative and **q**uantitative MRI **a**nalysis Neuroradiological assessment

Aside from standard clinical reports, two investigators (JS and LV) independently analyzed MR images following the guidelines of the new radiological classification for lissencephaly (Di Donato et al., 2017). The following categories were assessed: (i) gradient of gyral malformation, (ii) grade of gyral malformation, (iii) cortical thickness and appearance, and (iv) non-cortical brain malformations. A “thin” cortex in lissencephaly was defined as a cortex thinner than 10 mm in the majority of areas across the entire telencephalon.

##### Quantitative MRI analysis and qualitative analysis of cortical surface

Digital imaging and communications in medicine (DICOM) MR images of patients with lissencephaly were converted to the analyze format and were segmented semi-automatically using the ITK-SNAP software^2^ into gray and WM, and cerebrospinal fluid (CSF). Afterward, we manually segmented the gray matter into cortical gray matter (CGM) and basal ganglia with the thalamus. DICOM MR images of age-matched control subjects were automatically segmented with the CIVET processing pipeline using a web-based portal, CBRAIN (Sherif et al., 2014). An example of the tissue classification and segmentation in lissencephaly and control subjects can be found in Supplementary Figure S1.

Based on the segmented images, individual hemispheric outer surfaces were extracted by the Automated Segmentation with Proximities algorithm (MacDonald et al., 2000). This algorithm was used to deform a spherical mesh onto smoothed images using a threshold of 0.5. The gyrification index (GI) was defined as the ratio of the area of a surface model to that of its convex hull (Zilles et al., 1988). Convex hulls were extracted using a built-in function of CIVET 1.1.10, which then allowed for the calculation of the convex hull area and GI.

**FIGURE 1:**
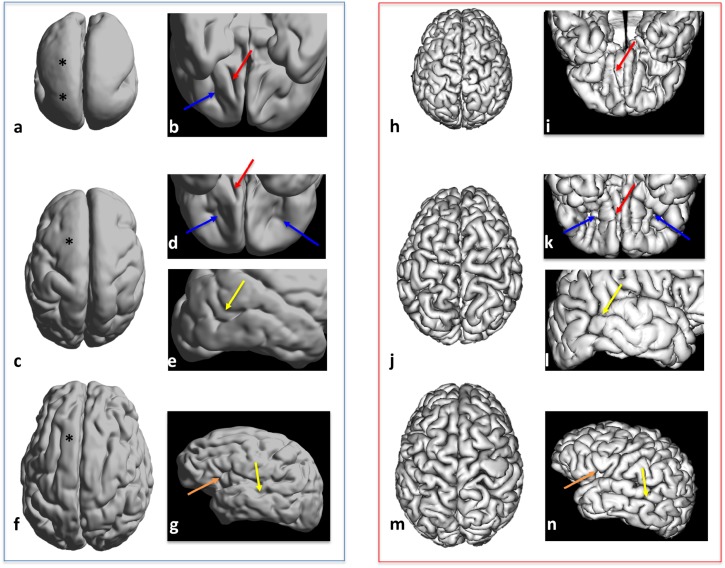
Three-dimensional views of cortical surface reconstruction of three subjects with lissencephaly: LIS_3 subject aged 5 years **(a,b)**, LIS_9 subject aged 2 years **(c–e)**, and LIS_8 subject aged 22 years **(f,g)** with age-matched controls **(h–n)**. Dorsal views **(a,c,f,h,j,m)**, ventral views **(b,d,i,k)**, and lateral views **(e,g,l,n)** are shown. Magnified areas showing normal-like simplified sulcal patterns in patients with lissencephaly and corresponding sulcal patterns in controls are shown in **b**, **d**, **e**, and **g:** olfactory sulcus (red arrow in **b**, **d**, **i**, and **k**), transverse orbital sulcus (blue arrow in **b**, **d**, and **k**), superior temporal sulcus (yellow arrow in **e**, **l**, **g**, and **n**), and ascending sulcus (ascending ramus of the lateral fissure, orange arrow in **g** and **n**). Areas of agyric cortex are marked by ^∗^.

Finally, on the reconstructed cortical surfaces (Figure 1), we identified gyri and sulci as well as the deepest parts of the sulci, so-called the sulcal pits (Lohmann et al., 2008) or roots (Régis et al., 2005).

##### Diffusion MRI analysis

Diffusion Toolkit and TrackVis^3^ were used to reconstruct and visualize tractography pathways. Tractography pathways were reconstructed using a HARDI model with a streamline/FACT algorithm and a 45° angle threshold. No threshold of fractional anisotropy (FA) was used for the fiber reconstruction. Brain mask volumes were used to terminate tractography structures instead of the standard FA threshold (Takahashi et al., 2012), because progressive myelination and crossing fibers in the developing brain can result in low FA values that may potentially incorrectly terminate tractography tracing in brain regions with low FA values.

The reconstruction of tractography pathways was performed using Diffusion Toolkit, and visualized with TrackVis^3^. A streamline algorithm for tractography was used for track reconstruction (Mori et al., 1999). A one-ROI approach was employed to reconstruct tracts with known anatomical landmarks (Catani and Thiebaut de Schotten, 2008). The following pathways were reconstructed both *in vivo* and *ex vivo* based on the methodology previously described (Vasung et al., 2017): basal forebrain and striatal pathways conveyed to the external capsule, pathways from the basal ganglia, thalamic pathways, and short-/long-range cortico-cortical pathways.

### Statistical Analysis

Metric data were described using minimum, maximum, mean, and standard deviation statistics for each group of subjects. Nominal data were described using absolute frequencies and percentages. Statistical analysis was performed using the SPSS^®^ software and estimationstats (Ho et al., 2018). Differences between the two groups in measured volumes, volume ratios, and GI for each hemisphere were assessed using a Student’s *t*-test. After the Bonferroni correction, a *p*-value ≤ 0.0038 was set as significant for all analyses. A linear mixed model for GI and for CGM/WM with group and hemisphere as factors, age, ventricular volumes, and interaction between group and age as covariates, and subject within a group as a random factor was used for which a *p*-value of ≤0.05 was considered significant.

#### Histology

After successful MR scans, the coronal slabs were sectioned and stained with neurofilament-specific antibody (SMI-312), which has been used to stain axonal fibers in immature fetal brains. Imaging was performed using a Nikon Instruments *Eclipse Ti*-E Inverted Microscope.

## Results

Ten patients with lissencephaly were scanned. Patient characteristics, patient age at scan, and MRI findings additional to lissencephaly were described in Supplementary Table S2.

### Qualitative Analysis of Structural MRI

The distribution of gyral malformations, agyria or pachygyria, and a “thin” or “thick” cortex was described in Supplementary Table S3. Non-cortical malformations were dysgenesis of the corpus callosum (*N* = 4), microcephaly (*N* = 4), ventriculomegaly (*N* = 3), and subcortical band heterotopia (*N* = 2) (Supplementary Table S2 and Supplementary Figures S1–S5). The cerebellum appeared properly foliated in all patients.

### Quantitative MRI Analysis

Quantitative MR measurements of the hemispheric GI and volumes of the third and lateral ventricles, hemispheric CGM, subcortical gray matter, hemispheric WM, the ratio between volume of the CGM and WM for 40 hemispheres across lissencephaly (*n* = 20 hemispheres) and control subjects (*n* = 20 hemispheres) are reported in Table 1.

**Table 1:** Age and hemispheric measurements in lissencephaly and control group.

	Lissencephaly					Controls					*p*-value
	*N*	Min	Max	Mean	*SD*	*N*	Min	Max	Mean	*SD*	
Age	10	0.68	21.67	6.26	6.51	10	0.86	20.05	6.11	6.07	0.95
GI_Left	10	1.03	1.17	1.08	0.04	10	2.12	3.01	2.74	0.27	*<0.01^∗^*
GI_Right	10	1.01	1.21	1.09	0.06	10	2.22	2.94	2.75	0.21	*<0.01^∗^*
Ventricles left	10	13,017.00	237,649.00	81,129.78	80,783.43	10	3,945.00	21,638.00	7,943.43	5,129.07	0.02
Ventricles right	10	13,228.00	201,941.00	71,078.47	65,915.27	10	4,207.00	19,589.00	8,592.5	4,416.74	0.01
CGM_Left	10	65,287.30	354,198.00	215,739.54	94,480.92	10	424,648.0	544,020.00	484,009.9	42,864.53	*<0.01^∗^*
CGM_Right	10	63,989.00	373,216.00	221,097.52	101,013.94	10	431,422.0	559,663.00	495,562.3	42,478.64	*<0.01^∗^*
SGM_Left	10	7,793.00	45,786.80	18,220.65	11,165.51	10	20,862.00	34,564.50	26,969.33	4,345.12	0.03
SGM_Right	10	10,916.40	47,229.50	19,841.41	10,966.65	10	20,658.00	34,531.60	27,504.41	4,288.72	0.05
WM_Left	10	20,541.20	214,531.00	91,193.95	68,717.74	10	213,669.00	286,442.00	255,168.0	20,860.47	*<0.01^∗^*
WM_Right	10	16,410.30	217,379.00	90,563.87	67,177.94	10	207,359.0	285,933.00	250,033.2	23,458.08	*<0.01^∗^*
CGM/WM left	10	1.31	4.58	2.9	1.08	10	1.48	2.48	1.92	0.3	0.01
CGM/WM right	10	1.48	4.19	2.93	0.97	10	1.51	2.49	2.0	0.3	0.01
Thick_cortex	4										

*Volumes are given in mm^3^. Thick cortex was assigned if a patient with lissencephaly had the cortical gray matter thicker than 10 mm on a coronal slice. After the Bonferroni correction significance level of the p was set to 0.0038. Significant differences of the means are in italics and are marked by ^∗^. GI, gyrification index; CGM, cerebral gray matter; WM, white matter; SD, standard deviation; SGM, subcortical gray matter including basal ganglia and thalamus.*

**FIGURE 2:**
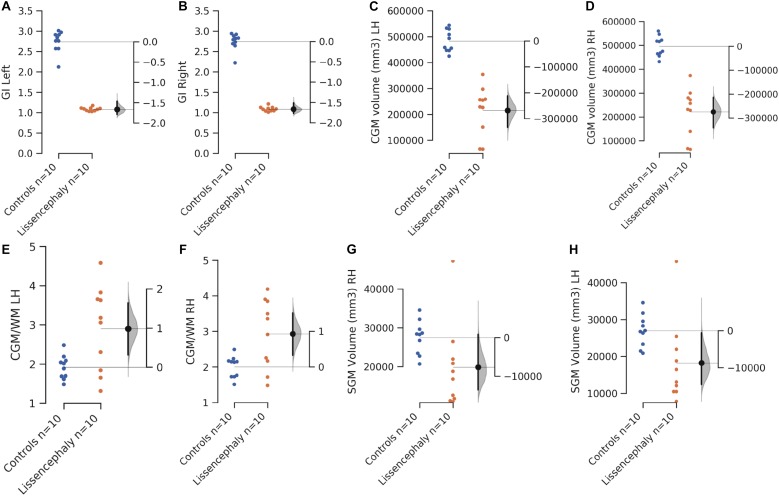
Scatterplots showing the comparison of the mean left and right hemispheric gyrification index **(A,B)**, CGM volume **(C,D)**, CGM/WM volume ratio **(E,F)**, and SGM volume **(G,H)** between controls (blues) and patients with lissencephaly (orange). The *y*-axis on the left side of each scatterplot shows the measured values. The shorter *y*-axis on the right is to show the difference of the means. The filled gray curve on the right of each scatterplot indicates the complete distribution of the effect size (difference of the means, Δ). The black line on the right of each scatterplot illustrates the 95% confidence interval of the Δ.

Hemispheric volumes of WM, CGM, and GI were significantly smaller in the lissencephaly group, compared to the control group (Figure 2 and Table 1). However, there was no significant difference between groups in the volumes of the subcortical gray matter (Figure 2 and Table 1).

The ratio of CGM/WM was not significantly different between hemispheres when controlling for age and pathological status. In addition, no significant correlation between ventricular volume and CGM/WM was found. This was tested because the presence of ventriculomegaly with hydrocephalus could produce the pressure to the WM and reduce its volume. CGM/WM was larger in the lissencephaly group, compared to the control group, after controlling for the volume, hemisphere, and age (Table 2). In addition, age-related decline in CGM/WM was significantly faster in the lissencephaly group (Table 2).

**Table 2:** Parameter estimates for linear mixed model of cortex/white matter volume ratio.

	*B*	95% CI		*p*-value^a^
Lissencephaly_group	1.74	1.17	2.32	**<0.001**
Left_hemisphere	-0.04	-0.14	0.06	0.38
Age	-0.04	-0.09	0.006	0.08
Ventricular_volume	<0.00	<0.00	0.00	0.051
Lissencephaly ^∗^ age	-0.1	-0.16	-0.03	**0.006**
Patient_(group)	0.15			

*^a^Linear mixed model with group and hemisphere as factors, age, ventricular volumes, and interaction between group and age as covariates, and subject within a group as a random factor. B, correlation coefficient. Significant p-values are in bold.*

### Qualitative Analysis of Cortical Surface

Reconstructed cortical surfaces demonstrated that despite the presence of agyria or pachygyria, certain cortical regions showed a relatively normal pattern of primary gyri and sulci (Figure 1). LIS_1 and LIS_2 patients had Sylvian fissures and well-developed deep sulcal pits of the superior frontal sulcus. LIS_2 had well-developed bilateral superior temporal sulci at the first time-point (1st year), but without secondary and tertiary sulci. However, during the first 2 years of life, morphological changes of these sulci were very discrete. LIS_3 patient had well-developed primary sulci [Figure 1a,b: transverse orbital sulcus (blue arrow); olfactory sulcus (red arrow)], as well as a definitive calcarine fissure and Sylvian fissure, and anterior deep sulcal pits of the parahippocampal and cingulate gyri. LIS_4 had detectable, normal-like shallow primary sulci of the frontal lobe. LIS_5 had a seemingly normal cingulate sulcus and identifiable sulcal pits of the anterior parahippocampal sulcus. LIS_6 had well-developed central and cingulate sulci. LIS_7 had primary and secondary sulci developed across the cerebral cortex; however, tertiary sulci were missing or appeared simplified (especially in frontal regions). LIS_8 had a simplified pattern of gyri and sulci, with all of the primary sulci [Figure 1f,g: superior temporal sulcus (yellow arrow)] and some secondary well-developed sulci [Figure 1f,g: ascending ramus of the lateral fissure (orange arrow)]. LIS_9 had a simplified pattern of primary gyri and sulci [Figure 1c–e: transverse orbital sulcus (blue arrows); olfactory sulcus (red arrow)], and few developed secondary sulci across the cerebral cortex (except in the frontal regions) [Figure 1c–e: superior temporal sulcus (yellow arrow)]. LIS_10 had a very shallow and undulating appearance of sulci across the entire cortex, making it difficult to distinguish between primary and secondary sulci.

### Qualitative Analysis of *ex vivo* Histology and *in vivo* DTI

**FIGURE 3:**
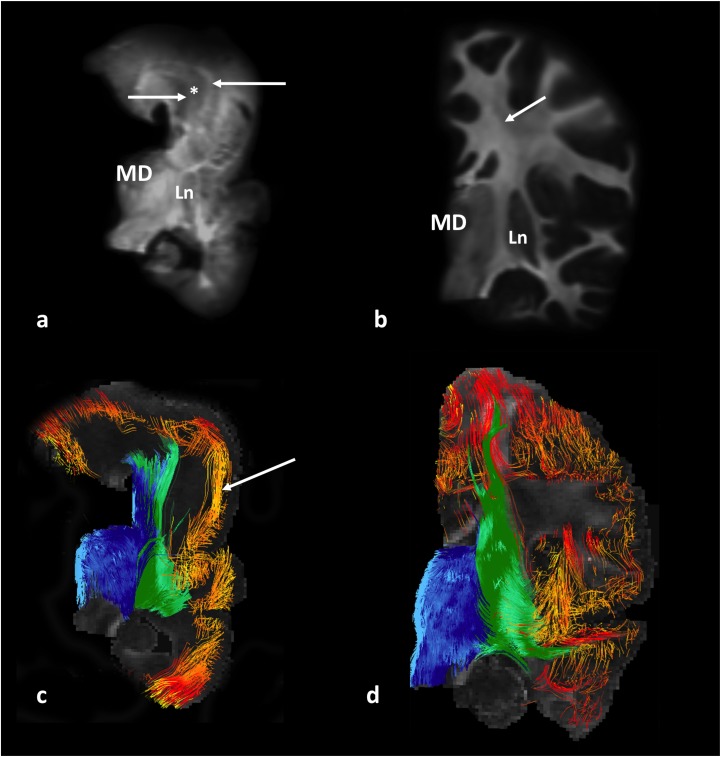
Diffusion-weighted MR images (upper row) and tract reconstructions (lower row) of post-mortem 1-year-old brain, specimen No. 641. Coronal slab passing through the mediodorsal nucleus of the thalamus (MD) with lissencephaly **(a,c)**, and an age-matched control, specimen No. 1798 **(b,d)** are shown. Arrows in **(a)** indicate areas of identified projection pathways (that normally pass through fiber crossing area) next to a hypo-intense area (^∗^). Arrow in **(b)** indicates a fiber crossing area, composed of callosal, projection, and association fibers. The composition of fibers that pass through this crossing area and originate from the thalamus (blue) and lentiform nucleus and basal forebrain fibers (green) can be seen in **c** and **d**. Note the rostro-caudal orientation of “cortical fibers of undetermined origin/ending” (in red/yellow) in the prospective area of the inferior frontal gyrus in the brain with lissencephaly (arrow in **c**). MD, mediodorsal nucleus of the thalamus; Ln, lentiform nucleus.

Qualitative analysis and DTI tract reconstructions revealed an altered spatiotemporal composition of projection pathways in lissencephaly (Figure 3), i.e., thalamocortical (Figure 3a,c, blue) and basal ganglia (Figure 3a,c, green) pathways appeared segregated in a crossing fiber-rich area (Figure 3a, asterisk) compared to controls (Figure 3b,d). Furthermore, our results revealed that compared to the normal age-matched brains (Figure 3d, orange–red pathways), brains with lissencephaly had a reduced amount of association pathways (Figure 3c, orange–red pathways) that showed altered, tangential orientations (Figure 3c, arrow).

Brain slices with neurofilament staining were processed and compared with tractography results in order to confirm that the reconstructed tractography pathways correspond to axonal fibers in lissencephaly and in control tissue slabs (Supplementary Figures S2, S3).

Finally, longitudinal *in vivo* analysis of DTI images of a subject with lissencephaly revealed altered fiber-architecture at two different time points: newborn and 5 years old (Supplementary Figure S4). Intra-cortical pathways placed in upper cortical layers showed tangential orientation (Supplementary Figure S4c, white arrow) while those in lower cortical layers showed radial orientation (Supplementary Figure S4c, black arrow). At both time points, we were not able to reconstruct pathways that connected cortical regions over a short distance (Supplementary Figure S4). Nevertheless, the inferior fronto-occipital fasciculus [Supplementary Figure S4d (in purple)] was reconstructed at both time points. Lastly, projection fiber bundles [Supplementary Figure S4d, thalamic fibers (in blue), basal ganglia, and basal forebrain fibers (in green)] continued to increase in volume with age.

## Discussion

Morphology and fiber pathways in the brain were examined using structural and diffusion MRI of patients with lissencephaly and neurologically healthy subjects. As could be expected, there was a significant difference in the hemispheric GI between the lissencephaly and age-/gender-matched control group (lissencephaly < control). In our dataset, the spatial distribution of agyric cortical regions was different in patients with *LIS1* (PAFAH1B1 gene) and *DCX* mutations. *LIS1* mutation was linked to diffuse agyria or predominant posterior regions of agyria, while *DCX* mutation predominantly affected anterior regions. Patients with lissencephaly had a preserved normal-like appearance of fissures and primary sulci in certain brain regions. Although patients with lissencephaly had significantly smaller hemispheric CGM and WM volume, we did not found significant differences between the groups in the volumes of the SGM. The patients with lissencephaly had a preserved spatiotemporal distribution of projection pathways, while short- to medium-range cortico-cortical association pathways were absent or fewer in number. Only a few detected cortico-cortical pathways in patients showed aberrant tangential trajectories within superficial cortical layers. There were also a number of radially oriented pathways within the lower cortical layers in patients with thick cortex-type lissencephaly, which were not present in control brains at the same age range. Lastly, we found a significant difference between the groups in the association between the age and CGM/WM volume ratio.

### Global and Local Gyrification

Although the mechanism for controlling global gyrification remains unclear, the majority of researchers suggest that there is a strong relationship between brain volume and the degree of gyrification (Im et al., 2008). However, others revealed, by a mathematical approach, that the absolute brain size plays a minor role in gyrification, and the length/number of fiber connections and the degree of gyrification are directly related across mammalian species (Herculano-Houzel et al., 2010). In agreement with the previous research, Germanaud et al. (2014) recently showed that there is a strong and positive relationship between the brain size and the level and complexity of gyrification and that this relationship can be characterized by allometric scaling laws for the general population. Moreover, using only the allometric scaling laws, one is able to fully explain the simplified gyral pattern and its variations in typically developing children with the smaller brain size. Contrarily, although these allometric scaling laws can be applied on children with brain development disorders (e.g., fetal alcohol syndrome or microcephaly), the gyrification complexity in these children might be unpredictable, i.e., disease-specific (Germanaud et al., 2014). Thus, the research from Germanaud et al. (2014) suggests that in the presence of brain development disease measurements of the brain size only might not be enough to predict the complexity of gyrification. Therefore, additional quantitative measures of the brain structures, such as volumes of the cerebral cortex, WM, and subcortical gray matter might be highly relevant for the identification of disease-specific gyrification pattern. In our study, we have measured volumes of the CGM, WM, and SGM in controls and patients with lissencephaly. Patients with lissencephaly had significantly smaller volumes of both CGM and WM and reduced gyral complexity. Although our results are in agreement with the previous research suggesting the link between gyrification and brain size, the smaller volumes of CGM and WM found in patients with lissencephaly might indicate the relevance of proper WM connectivity and cortical growth in the process of formation of cortical convolutions.

Patients with lissencephaly showed a negative association between the CGM/WM volume ratio and age indicating disproportional growth of WM compared to the CGM with age. These findings, limited by small number of subjects, resemble to the findings in normal human fetuses in which, during the last two trimesters [the periods of peak gyrification (Vasung et al., 2016) and rapid increase in the number of cortico-cortical connections (Vasung et al., 2017)], growth of WM volume surpasses the growth of CGM. Given that patients with lissencephaly showed atypical spatiotemporal patterns of brain connectivity (preservation of the long-range connections while only a low amount of short connections were observed) these findings suggest that sequential spatiotemporal development of connectivity and proper maturation of cerebral cortex most likely play a role in the process of gyrification. Another theory suggests that regionally differential rates of tangential expansions of the CGM, predominantly influenced by different cytoarchitectonic patterns across brain regions, may cause a “pattern-specific folding” (Ronan and Fletcher, 2015). This differential tangential expansion is possibly related to an abundant outgrowth and ingrowth of axonal upper-layer association connections in the second and third trimesters (Kostoviæ and Jovanov-Miloseviæ, 2006; Vasung et al., 2017). In addition, significant areal differences in gene expressions during the second and early third trimesters of pregnancy (Pletikos et al., 2014) might be linked to differential rates of tangential expansions between cortical areas. Therefore, one could speculate that the appearance of pattern-specific cortical folding is determined by conserved genetic mechanisms prenatally. Although we found significantly smaller volumes of CGM in lissencephaly, we did not assess regional differences in cortical volume between groups. Thus, lissencephaly, which is characterized by a reversed order of cortical layers, could provide a diseases model to test these hypotheses in the future.

The cerebellum appeared to be properly foliated in all patients. However, the variation in the voxel size and MRI quality limited the quantitative analysis of cerebellar folia. Involvement of cerebellum in lissencephaly has been associated with the mutations of DCX, TUBA1A, and LIS1 genes, and represents a distinctive heterogeneous group of cortical malformations (Ross et al., 2001). The severity of cerebellar abnormality in lissencephaly ranges from discrete midline hypoplasia to disturbed foliation and volume reduction (Ross et al., 2001), later being most commonly associated with the involvement the TUBA1A (Poirier et al., 2007) and RELN genes (Hong et al., 2000; Chang et al., 2007). RELN gene codes for extracellular matrix glycoprotein Reelin (Ogawa et al., 1995). During the fetal brain development, Reelin is secreted in telencephalon by Cajal–Retzius cells within the marginal zone [where it regulates neuronal migration (Tissir and Goffinet, 2003)] and in the cerebellum by the cells in the external granular layer (where it promotes Purkinje cell migration). Thus, the lack of cerebellar findings in our study could be explained by the lack of patients with identified RELN or TUBA1A mutations. However, this remains to be determined on a larger cohort of patients with lissencephaly.

### Brain Microarchitecture in Lissencephaly

The average volume of the cerebral cortex of the hemisphere in patients with lissencephaly was two and a half times smaller compared to age-matched controls, and all of our lissencephaly patients had thicker cerebral cortices than normal subjects (>5 mm) (Supplementary Table S3). This altered appearance of the cerebral cortex is most likely related to arrested neuronal migration and altered dendritic arborization of heterotopic pyramidal cells (Fleck et al., 2000). Our qualitative analysis of reconstructed fiber tracts in “thick” (Figure 3 and Supplementary Figures S2, S4) and “thin” (not shown) type of lissencephaly (Di Donato et al., 2017) revealed altered organization and orientation of cortical pathways, located or originating from superior cortical layers (Figure 3 and Supplementary Figure S4, white arrow), while projection pathways seem to preserve their proper orientation and position (Figure 3 and Supplementary Figure S4, white arrow) in both forms of lissencephaly.

In order to confirm whether tractography pathways correspond to axonal fibers, we have correlated our findings with histologically stained sections for neurofilament components. Our results revealed that patients with lissencephaly have altered architecture and orientation of axonal fibers, correlated with tractography pathways. Given that neuronal migration in lissencephaly is altered, especially of those neurons destined to upper cortical layers, improper position of neurons would ultimately lead to failure of proper development of short- to medium-range cortico-cortical association connections predominantly. These results are in agreement with other investigators (Poretti et al., 2013; Kamiya et al., 2014). However, due to the known limitations of diffusion tensor imaging (Jones et al., 2013), a larger study with co-registered histological slices is needed in order to characterize intracortical fibers in patients with various types of lissencephaly.

Finally, the negative association between age and CGM/WM volume suggests that during the development WM, compared to the CGM, grows faster in patients with lissencephaly. In addition, patients with lissencephaly had altered architecture of WM fibers, mostly affecting association fibers connecting upper cortical layers between different cortical areas. As patients with lissencephaly did not show a significant difference in the volume of SGM we suggest that the disproportionate age-related increase in the WM, compared to the CGM, could most likely reflect the age-related increase in the volume of projection fiber system. Thus, our results support the idea that detailed analysis of connectivity in patients with lissencephaly may be useful in future for classification of lissencephaly in relation to the clinical outcome, which has not been shown with the existing lissencephaly classifications (Di Donato et al., 2017). Moreover, given that myelination is somewhat preserved in lissencephaly (Barkovich et al., 1991) and that early brain activity is related to brain volume growth (Benders et al., 2015), about one-third of which is occupied by the WM volume, it is possible that an accelerated growth of the WM in patients with lissencephaly offers a window of opportunity for functional maturation and rehabilitation of survivors. However, this remains to be tested in a larger homogenous group of patients with this disorder.

### Limitations

Although all patients showed some common brain features in this study regardless of the genetic heterogeneity, the small sample size, differences in MRI acquisition parameters, and the genetic heterogeneity of the patients in this series may limit the power of the study with potential confounding. Lastly, due to the limited *ex vivo* tissue material (tissue blocks), we were not able to identify with certainty specific fiber pathways (such as inferior fronto-occipital fascicle or cerebrospinal tract).

## Conclusion

Our results indicate that the severity and spatial distribution of agyria in patients with lissencephaly tend to be linked with identified genetic abnormalities (*LIS1* or *DCX* mutation). Furthermore, patients with lissencephaly had significantly smaller volumes of the white and CGM compared to age-matched control groups. However, there was no significant difference between the groups in the volumes of the SGM. Histological and postmortem MR analyses indicated preserved spatiotemporal distribution of projection pathways, but the absence or few in number of short- to medium-range cortico-cortical association pathways in patients with lissencephaly. Thus, our results indicate that in patients with lissencephaly cortical system is affected more than the subcortical one.

## Ethics Statement

Since this study was retrospective in nature and no identifying information would be requested by the researchers, it was determined that the informed consent would not be sought from the patients due to the low risk this study would present to them. Thus, a waiver of consent, in addition to all other procedures, was approved after due consideration by the BCH Institutional Review Board.

## Author Contributions

ET, AvdK, and NS collected the data. LV, AR, HY, JS, AP, FC-D, and ET analyzed the data. LV, TS, JL, and ET wrote and edited the manuscript.

## Conflict of Interest Statement

The authors declare that the research was conducted in the absence of any commercial or financial relationships that could be construed as a potential conflict of interest.

## Footnotes

1 http://medschool.umaryland.edu/BTBank/

2 http://www.itksnap.org

3 http://www.trackvis.org
