# Supplementary material for: Structural and Diffusion MRI Analyses With Histological Observations in Patients With Lissencephaly
Source: Front Cell Dev Biol. 2019 Jul 11;7:124. doi: 10.3389/fcell.2019.00124 (PMC6637974; doi:10.3389/fcell.2019.00124)
Supplement: Supplementary file 1 [file Data_Sheet_1.docx]

**Supplementary information**

| **Table S1.** MRI parameters used during the *in vivo* MRI acquisition with the age of the patient during scan. | | | | | | |
| --- | --- | --- | --- | --- | --- | --- |
| **Patient** | **1st MRI** | **Age (years)** | **2nd MRI** | **Age**  **(years)** | **3rd MRI** | **Age**  **(years)** |
| LIS_1 | 3T, T1 MPR, 256x256, 0.78x0.78x0.9mm  ___________  **DTI: 35 directions, b=1000, 1.71x1.71x2 mm** | 4.91 |  |  |  |  |
| LIS_2 | 1.5T, T1 MPR, 512x384, 0.35x0.35x2mm | 0.7 | 1.5T, T1 MPR, 256x256, 0.70x0.70x1.5mm | 2.36 |  |  |
| LIS_3 | 3T, T2 TSE COR, 320x320, 0.56x0.56x4mm  ___________  **DTI: 12 directions, b=1000, 1.95x1.95x4 mm** | 0.24 | 3T, T1 MPR, 192x192, 1x1x1mm  ___________  **DTI: 35 directions, b=1000, 1x1x2 mm** | 4.57 |  |  |
| LIS_4 | 1.5T, T1, 256x224, 0.78x0.78x3mm | 0 | 1.5T, T1, 256x256, 0.78x0.78x4mm  ___________  **DTI: 6 directions, b=1000, 0.94x0.94x3 mm** | 0.32 | 1.5T, T1 C+, 320x224, 0.39x0.39x5mm | 3 |
| LIS_5 | 1.5T, T2 FSE Axial, 256x256, 0.78x0.78x3mm | 0 | 1.5T, T1, matrix size: 256x256, 0.86x0.86x1.5mm | 5.55 | 1.5T, T1 MPRAGE, 256x256, 0.82x0.82x0.9mm  ___________  **DTI: 35 directions, b=1000, 2x2x2 mm** | 10.6 |
| LIS_6 | 1.5T, T1 MPR SAG, 256x256, 0.85x0.85x5mm | 0.86 |  |  |  |  |
| LIS_7 | 3T, T1 MPR, 256x256, 0.78x0.78x0.9mm  __________  **DTI: 30 directions, b=1000, 2x2x2 mm** | 11.18 |  |  |  |  |
| LIS_8 | 3T, T1 MPR, 244x244, 0.75x0.75x1mm  ___________  **DTI: 35 directions, b=1000, 2x2x2 mm** | 21.67 |  |  |  |  |
| LIS_9 | 3T, T1 MPR, 192x192, 0.70x0.70x1mm  ___________  **DTI: 35 directions, b=1000, 2x2x2 mm** | 1.57 |  |  |  |  |
| LIS_10 | 3T, T1 MPR, 256x256, 0.78x0.78x0.9mm  ___________  **DTI: 30 directions, b=1000, 2x2x2 mm** | 2.11 |  |  |  |  |
| ***MR imaging*** *parameters are reported in the following order;* ***Field strength*** *(3T or 1.5T),* ***Sequence (****(T1 multi-planar reconstruction (T1MPR), T1-weighted magnetisation-prepared rapid gradient-echo (T1 MPRAGE), T1 with gadolinium contrast (T1 C+), T2 coronal turbo-spin-echo (T2 TSE COR), T2 axial fast spin echo (T2 FSE Axial)),* ***Matrix size, Voxel size*** *(X*Y*slice thickness in mm).*  ***DTI*** *imaging parameters are reported in bold in following order:* ***DTI*** *(diffusion tensor imaging),* ***Number of directions****,* ***b value****,* ***Voxel size*** | | | | | | |

| **Table S2.** Patient characteristics, patient age during scan(s), and MRI findings additional to lissencephaly | | | | | | | | |
| --- | --- | --- | --- | --- | --- | --- | --- | --- |
| **Patient** | **Age at MRI scan (years)** | | **Additional diagnosis/genetic abnormality** | | **Additional MR findings** | | | |
| LIS_1 | 4.91 | | - | | Microcephaly, bilateral malrotation of hippocampi, ventriculomegaly | |  |  |
| LIS_2 | 0.7, 2.36 | | No identifiable genetic abnormality | | Thin corpus callosum, bilateral malrotation of hippocampi, microcephaly, ventriculomegaly | |  |  |
| LIS_3 | 0.24, 4.57 | | Miller Deiker Sy  **LIS1 deletion** | | Hypoplasia of gene and anterior body of the corpus callosum, T2 hyperintensities adjacent to the frontal horns | |  |  |
| LIS_4 | 0, 0.32, 3 | | Lissencephaly II | | Agenesis of the corpus callosum, vermian hypogenesis, ventriculomegaly, Dandy Walker, microcephlay | |  |  |
| LIS_5 | 0,5.55, 10.6 | | Muscle-eye-brain disease | | Cobblestone lissencephaly, thin corpus callosum, hypoplasia of vermis and brainstem, left temporoparietal cyst | |  |  |
| LIS_6 | 0.86 | | **LIS1 mutation** | | Periventricular nodule of subcortical heterotopia | |  |  |
| LIS_7 | 11.18 | | G6PD | | Microcephaly, periventricular gliosis, microcephaly | |  |  |
| LIS_8 | 21.67 | | Lennox Gastaut syndrome, Charcot Marie Tooth | | Heterotopic periventricular and subependymal gray matter and within the white matter, smaller left cerebellar hemisphere | |  |  |
| LIS_9 | | 1.57 | | DCX lissencephaly | | Hypogenesis of basal ganglia and brain stem | | |
| LIS_10 | | 2.11 | | DCX mutation | | Band heterotopia | | |
| Lissencephaly-1 gene (LIS1). Doublecortin (DCX). Glucose 6 phosphate dehydrogenase deficiency (G6PD). The order of patients is based on the order of admission at the hospital. | | | | | | | | |

| **Table S3.** *In vivo* MR readings of patients with lissencephaly according to Di Donato et al., 2017. | | | | |
| --- | --- | --- | --- | --- |
| **Patient** | **Additional diagnosis/genetic abnormality** | **Gradient of gyral malformation** | **Grade of gyral malformation** | **Cortical thickness/appearance** |
| LIS_1 | - | Temporal more severe | Agyria-pachygyria | “Thin” mixed disgyria |
| LIS_2 | No identifiable genetic abnormality | Posterior more severe | Agyria-pachygyria | “Thin” variable disgyria |
| LIS_3 | Miller Deiker Sy  LIS1 (de novo deletion at 17p13.3) | Diffuse | Diffuse agyria | “Thick” classic |
| LIS_4 | Muscle-eye-brain disease most likely because giant visually evoked potentials Lissencephaly II | Posterior more severe | Agyria-pachygyria | “Thin” variable disgyria |
| LIS_5 | Muscle-eye-brain disease of unclear etiology, a single variant in a COL18A1 gene with the second variant not found | Diffuse | Diffuse agyria | “Thin” undulating |
| LIS_6 | LIS1 mutation (C.1193G3A change in LIS1 gene), additional deletions at 18q22.1 and 1q21.1 | Posterior more severe | Diffuse agyria | “Thick” classic |
| LIS_7 | G6PD incomplete lissencephaly | Anterior more severe | Partial pachygyria | “Thin” variable disgyria |
| LIS_8 | Duplication of chromosome 17p12 (containing the PMP22 gene), Lennox Gastaut syndrome, Charcot Marie Tooth | Anterior more severe | Partial pachygyria,  Simplified gyration overlying SBH | Simplified gyration overlying “thin” SBH and variable SBH |
| LIS_9 | DCX lissencephaly (586 C>T substitution=resulting in arginine 196 to cysteine missense mutation) | Anterior more severe | Agyria-pachygyria | “Thick” classic |
| LIS_10 | heterozygous mutation of C.176G>A = x-linked lissencephaly or double cortex syndrome DCX mutation (176 G>A) | Diffuse | SBH diffuse | Simplified gyration overlying “thick” SBH (13mm) |

| **Table S4**. Characteristics of patients used for *ex-vivo* analysis (age and cause of death, pathological, and gene abnormality findings additional to lissencephaly). Specimen number is based on the number provided by the Brain Bank. | | | | | | | | |  |  |  |
| --- | --- | --- | --- | --- | --- | --- | --- | --- | --- | --- | --- |
| Specimen number | Age | Sex | Cause of death | Brain malformations | | Gene abnormality | | Lissencephaly characteristic |  | |  |
| 1164 | 19 years and 313 days | Male | Complications of Disorder | Lissencephaly | None identified | | Pachygyria/lyssencephaly, thick cortex (1.4 cm) sparing temporal lobes with radially oriented disorganized neurons, decreased volume of white matter, descending pyramidal tract degeneration | |  |  | |
| 4524 | 13 years | Male | Complications of Disorder | Lissencephaly | No report | | Pachygyria-agyria, thick cortex | |  |  | |
| 683 | 8 years and 306 days | Female | Complications of Disorder | Lissencephaly type I, Agenesis of corpus callosum, microcephaly | None identified | | Lissencephaly type I (predominant posterior), pachygyria, thick cortex (4mm superficial layers, 2cm deeper cortical band), focal gray matter heterotopias | |  |  | |
| 641 | 1 year and 163 days | Female | Complications of Disorder | Lissencephaly type I, Johanson-Blizzard syndrome, Miller-Deiker syndrome | DELETION of 17p13 | | Lissencephaly type I with pachygyria, thick cortex (2cm), gray matter heterotopia | |  |  | |
| 1841 | 19 years and 289 days | Male | Multiple injuries | Control |  | |  | |  |  | |
| 5376 | 13 years and 101 days | Male | Hanging | Control |  | |  | |  |  | |
| M3835M | 9 years and 224 days | Female | Asphyxia | Control |  | |  | |  |  | |
| 1798 | 1 year and 288 days | Female | Intussusception | Control |  | |  | |  |  | |


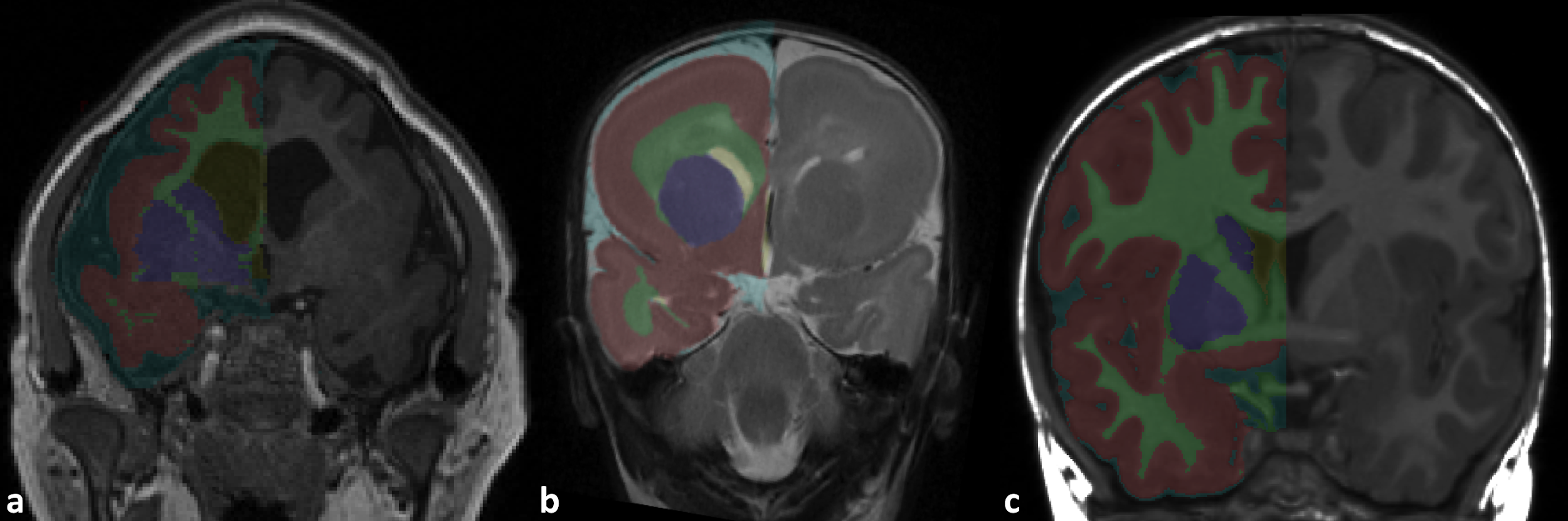


**Figure S1**.

An example of semi-automatic hemispheric tissue classification of the T1-weighted brain MRI from a five-year-old patient (subject LIS_1) with lissencephaly (a), T2-weighted brain MRI from an infant (subject LIS_3) with lissencephaly (b), and T1-weighted brain MRI from a five-year age-matched control subject (c). The MRIs were segmented into the background (subarachnoid space in sea blue), cerebral cortex (red), white matter (green), basal ganglia with the thalamus (blue), and ventricles (yellow).


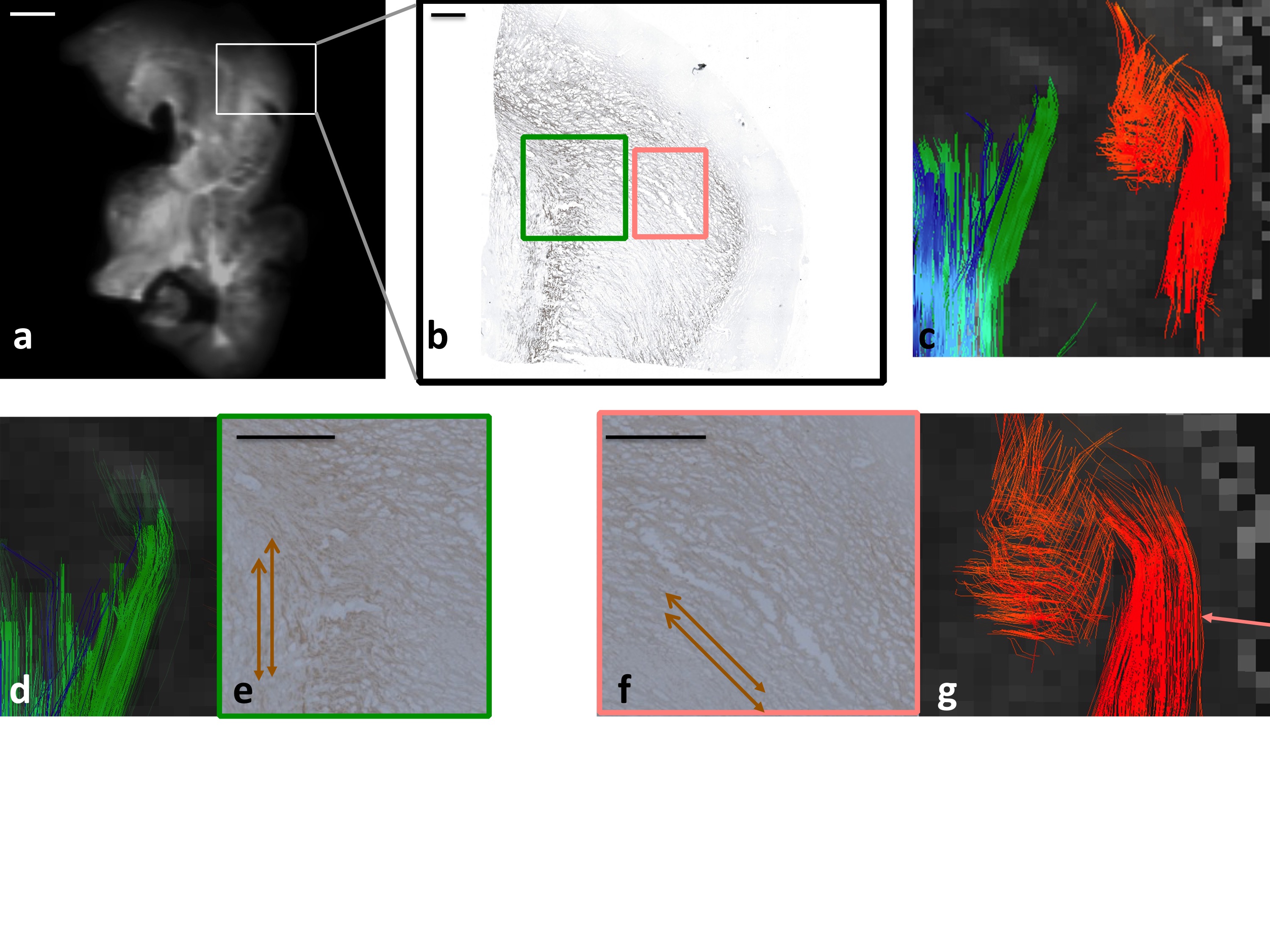


**Figure S2.**

Diffusion-weighted MR image of a coronal slab of a 1-year-old brain with lissencephaly, specimen No. 641, (a) with the corresponding neurofilament stained histological section (b, a rectangular area in a), and with the reconstruction of tracts that pass through the same rectangular area (c). Magnification of the histological section marked by rectangular green area in b is shown in panel e. Arrows in e show the orientation of the neurofilaments. Tract reconstruction in d shows basal forebrain, pathways from the lentiform nucleus (green), and thalamic pathways (blue) passing through these regions. Magnification of the histological section marked by rectangular salmon area in b is shown in panel f. Arrows in f show the orientation of the neurofilaments. Tract reconstruction in g shows cortical fibers oriented radially and tangentially (arrow) without identified origins or terminal areas. The scale bar in (a) is approximately 10mm wide while in figures (b-g) it is 1mm wide.


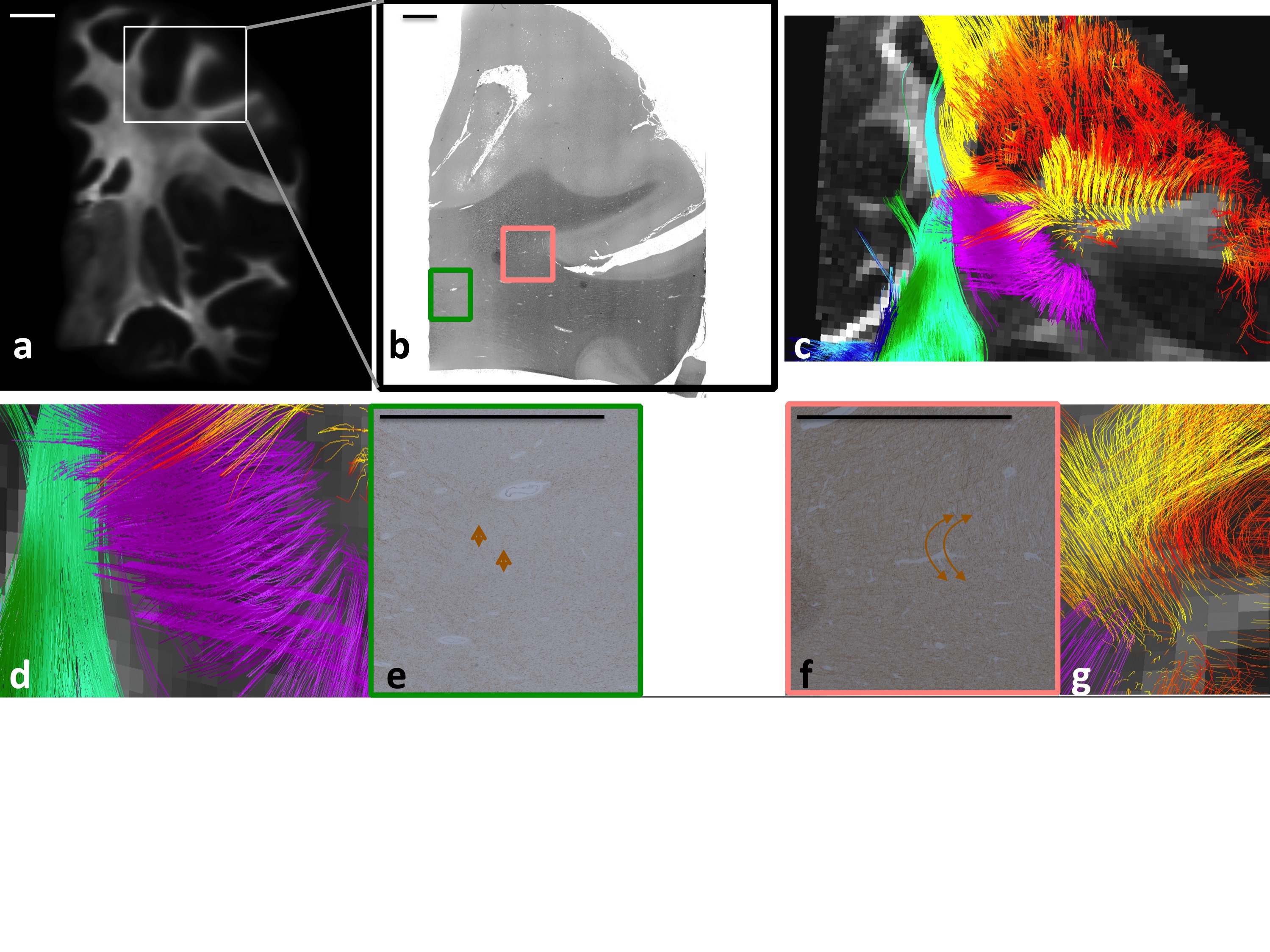


**Figure S3.** Diffusion-weighted MR image of a coronal block of a 1-year-old **control** brain, specimen No. 1798, (a) with the corresponding neurofilament stained histological section (b), a rectangular area in (a), and with the reconstruction of tracts that pass through the same rectangular area (c).

Magnification of the histological section marked by rectangular green area in (b) is shown in panel (e). Arrows in (e) show the orientation of the neurofilaments (sparse areas indicating fronto-occipital orientation). Tract reconstruction in (d) shows basal forebrain, fibers from the lentiform nucleus (green), and long associative areas (purple) passing through these regions.

Magnification of the histological section marked by rectangular orange area in (b) is shown in panel (f). Arrows in (f) show the orientation of the neurofilaments. Tract reconstruction in (g) shows cortical fibers without identified origin/end’. However, it is clear that the majority of these fibers are composed of fibers with a U-shape. The scale bar in (a) is approximately 10mm wide while in figures (b-g) it is 1mm wide.

* Note - nor long association (purple) nor short cortical U-fibers in this region of the brain weren't found in the previous brain with lissencephaly.


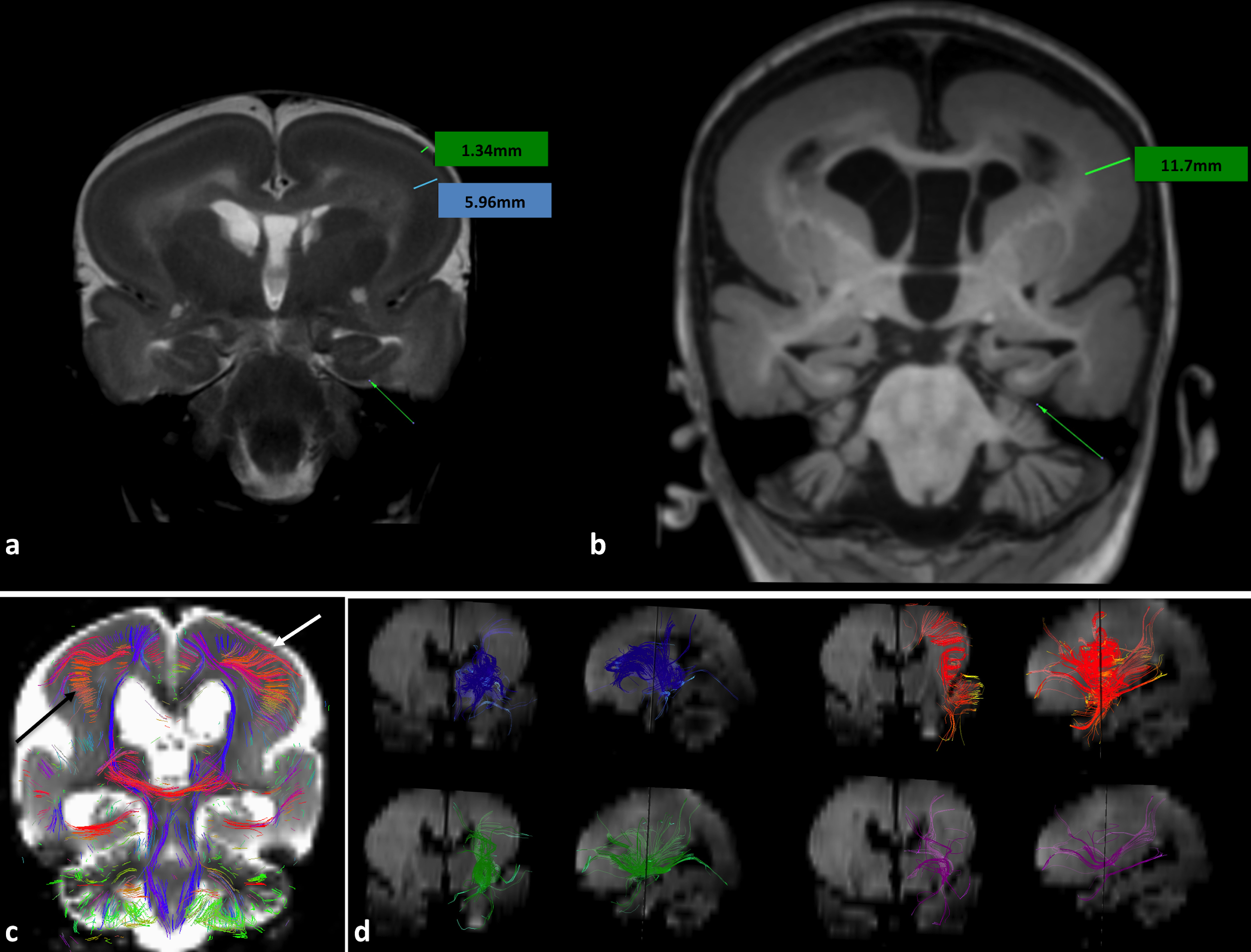


**Figure S4.**

**Upper row:** Coronal section of an *in vivo* acquired MR image of a child with lissencephaly (Subject LIS_3) during the 1st year of life (T2-weighted image (a)) and during the 4th year of life (T1-weighted image (b)). The images are not scaled by size. However, measurement of the cortical layers can be found in the blue and green boxes (a, b). Note the proper rotation of hippocampi (a, b, green arrows).

**Lower rows:** Diffusion-weighted image (DWI) with the superimposed reconstructed fiber pathways during the 1st year of life (c) and DWI with the superimposed reconstructed fiber pathways during the 4th year of life (d).

Note the tangential fibers located in the superficial cortical layers (white arrow in c) and radial streamlines in lower and thicker cortical layers (black arrow in c). Thalamic pathways (blue in d), basal ganglia and basal forebrain pathways (green in d), inferior fronto-occipital fasciculus (purple in d), and cortical radial fibers of undetermined ending/origins (red in d).


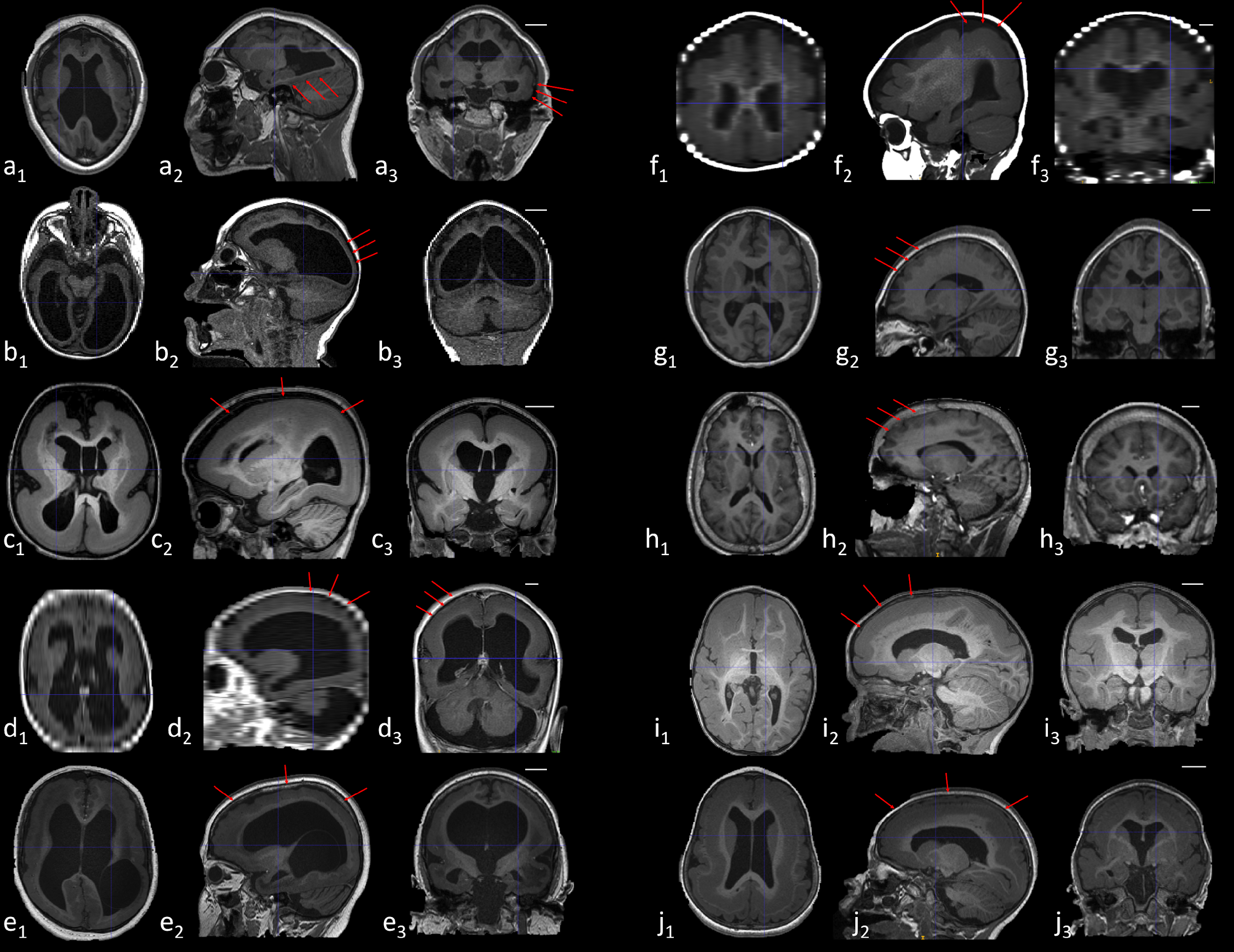


**Figure S5.**

Axial (_1_), sagittal (_2_), and coronal (_3_) MRI of LIS_1 (a), LIS_2 (b), LIS_3 (c), LIS_4 (d), LIS_5 (e), LIS_6 (f), LIS_7 (g), LIS_8 (h), LIS_9 (i), and LIS_10 (j) subjects. Triple red arrows indicate an area where agyria or simplified gyral pattern predominates. The scale bar at the upper right corner of the coronal images is approximately 10mm wide.
